# Killing of Mycolic Acid-Containing Bacteria Aborted Induction of Antibiotic Production by *Streptomyces* in Combined-Culture

**DOI:** 10.1371/journal.pone.0142372

**Published:** 2015-11-06

**Authors:** Shumpei Asamizu, Taro Ozaki, Kanae Teramoto, Katsuya Satoh, Hiroyasu Onaka

**Affiliations:** 1 Department of Biotechnology, Graduate School of Agricultural and Life Sciences, The University of Tokyo, Bunkyo, Tokyo, Japan; 2 Advanced Technology Department, JEOL Ltd., Akishima, Tokyo, Japan; 3 Ion Beam Mutagenesis Research Group, Biotechnology and Medical Application Division, Quantum Beam Science Center, Japan Atomic Energy Agency, Takasaki, Gunma, Japan; Friedrich Schiller University, GERMANY

## Abstract

Co-culture of *Streptomyces* with mycolic acid-containing bacteria (MACB), which we termed “combined-culture,” alters the secondary metabolism pattern in *Streptomyces* and has been a useful method for the discovery of bioactive natural products. In the course of our investigation to identify the inducing factor(s) of MACB, we previously observed that production of pigments in *Streptomyces lividans* was not induced by factors such as culture extracts or mycolic acids. Although dynamic changes occurred in culture conditions because of MACB, the activation of pigment production by *S*. *lividans* was observed in a limited area where both colonies were in direct contact. This suggested that direct attachment of cells is a requirement and that components on the MACB cell membrane may play an important role in the response by *S*. *lividans*. Here we examined whether this response was influenced by dead MACB that possess intact mycolic acids assembled on the outer cell membrane. Formaldehyde fixation and γ-irradiation were used to prepare dead cells that retain their shape and mycolic acids of three MACB species: *Tsukamurella pulmonis*, *Rhodococcus erythropolis*, and *Rhodococcus opacus*. Culturing tests verified that *S*. *lividans* does not respond to the intact dead cells of three MACB. Observation of combined-culture by scanning electron microscopy (SEM) indicated that adhesion of live MACB to *S*. *lividans* mycelia were a significant interaction that resulted in formation of co-aggregation. In contrast, in the SEM analysis, dead cells were not observed to adhere. Therefore, direct attachment by live MACB cells is proposed as one of the possible factors that causes *Streptomyces* to alter its specialized metabolism in combined-culture.

## Introduction

We have investigated co-culture methods using bacterial interactions with the aim of activating cryptic secondary metabolite-encoding gene clusters, for natural product discovery in *Streptomyces* species. Methodologies aimed at triggering the expression of secondary metabolite-encoding cryptic biosynthetic gene clusters involve simulating the natural environment, which consists of complex microbial communities [1,2]. In the course of this study, we found that inter-generic interaction between *Streptomyces lividans* and *Tsukamurella pulmonis* TP-B0596 affected the production of secondary metabolites by *S*. *lividans* [3].

*Streptomyces* species are a common soil-dwelling filamentous bacterium and is well-known as a producer of clinically important bioactive secondary metabolites. Natural products derived from *Streptomyces* continue to play important roles in drug discovery [4]. Recent genome analyses of *Streptomyces* indicate the existence of 20–40 cryptic secondary metabolite biosynthetic gene clusters in a single strain [5], suggesting that only a small fraction of the diverse natural products have actually been isolated.

Activation of secondary metabolism by small molecules or environmental stresses has been investigated in detail [6,7]. Intra-species auto-inducers, γ-butyrolactones (e.g., A-factor), in *Streptomyces* species are known for morphological differentiation and regulation of secondary metabolism [8]. Other factors that lead to the activation of gene clusters encoding undecylprodigiosin (RED) or actinorhodin (ACT) in *Streptomyces coelicolor* A3(2) have also been well studied. For example, phosphate limitations are required for the production of many antibiotics [9]. *N*-acetylglucosamine up-regulates the production of RED and ACT in *S*. *coelicolor* in minimal medium condition [10]. Chemical elicitors such as PI factor [11], goadsporin [12,13], hormaomycin [14] and several synthetic compounds [15,16] are also found to affect secondary metabolism in *Streptomyces* species. More recently bacterial-bacterial [3] or bacterial-fungal [17] interactions have attracted more attention, since the interactions have been demonstrated to activate cryptic secondary metabolism in the bacteria or fungi involved [18]. *Bacillus subtilis* secreted surfactant-like molecules (e.g., bacillaene, surfactin) which inhibited aerial formation and production of RED in *S*. *coelicolor* A3(2) [19], whereas inhibition of those production in *B*. *subtilis* resulted in the induction of RED by *S*. *coelicolor* or *S*. *lividans* [20]. Interactions between *S*. *coelicolor* and several actinomycetes also resulted in the production of secreted siderophores, desferrioxamine derivatives, by *S*. *coelicolor*, indicating Fe competition [21]. Co-cultivation of *Aspergillus niger* and *S*. *coelicolor* resulted in finding new natural products from *A*. *niger* [22]. Unique experiments by heat-killed cells of *B*. *subtilis* and *Staphylococcus aureus* were also found to enhance production of RED in *S*. *coelicolor* [23,24]. While there are numerous reports on attempts to activate the production of cryptic secondary metabolites, reports proposing the direct interactions involved in the activation of secondary metabolism in microbes are still few in number [3,18]. Brakhage and co-workers showed that *Streptomyces rapamycinicus* directly attached to *Aspergillus nidulans* hypha during the activation of polyketide antibiotics biosynthesis [17]. The activation of fungal secondary metabolism occurs via Saga/Ada-mediated histone acetylation, which upregulates the expression of polyketide antibiotics biosynthetic genes [25]. These results suggest that, as an alternative to chemical communication [26,27], naturally occurring direct physical interactions form a fundamental means of communication between microbes. However, the mechanisms by which signals generated by physical attachment induce the activation of secondary metabolism in fungi are still unknown.

In our previous study, *S*. *lividans*, a conditional producer of two pigments (RED and ACT), was used as an indicator strain for screening applications. *T*. *pulmonis*, a mycolic acid containing bacterium (MACB) isolated from soil, was found to induce the production of both pigments in *S*. *lividans*. It was further found that *T*. *pulmonis* activated the production of cryptic antibiotics in a broad range of *Streptomyces* species, which resulted in the discovery of new bioactive natural products, e.g., alchivemycin A [28], arcyriaflavin E [29], chojalactones [30], and 5-alkyl-1,2,3,4-tetrahydroquinolines [31], and more recently, Bachmann and coworkers employed this combination to discover ciromicins [32]. Interestingly, this effect was induced by diverse genera of MACB, whereas typical microbes (e.g. *Bacillus subtilis*, *Staphylococcus aureus*, *Saccharomyces cerevisiae*, *Candida albicans*) did not show the inducing effects [3]. In view of the potency of the actinomycetes and MACB co-culture in the activation of cryptic secondary metabolism, we termed this method “combined-culture” [3].

MACB belong to the suborder *Corynebacterineae* and are members of a family of Gram-positive rod-shaped bacteria that includes the pathogenic bacterium *Mycobacterium tuberculosis* [33], the industrial glutamate producer *Corynebacterium glutamicum* [34], and *Rhodococcus* species, which decompose organic pollutants [35]. The outer membrane of MACB has a lipid-rich structure. Although the complete assembly of the cell membrane structure is not fully understood, in most well-studied MACB, the *Mycobacterium* outer membrane consists of glycopeptidolipids, triacylglycerols, diacylglycerols, trehalose 6,6′-dimycolates, and mycolic acids, as major components [36,37]. Of these outer membrane lipids, mycolic acids represent a specifically well-conserved component within the MACB.

To identify inducing factors in MACB, we previously tested extracts containing mycolic acids. However, they were not found to induce antibiotic production [3]. Subsequently, to search for diffused molecules produced by *T*. *pulmonis*, butanol extracts and membrane filtered culture supernatants were tested, but these did not show inducing activity either [3]. Additionally, to search for effects of culture condition changes (pH, ion, etc.) caused by *T*. *pulmonis*, dialysis (membrane separated vessel) flasks [38], which can separate two culture vessels of *T*. *pulmonis* and *S*. *lividans* by a dialysis membrane, were used, however *S*. *lividans* was not induced to produce pigments in this case as well [3].

These previous results were considered to rule out the possibility of known inducing factors for pigment production by *S*. *lividans*. Therefore, we predicted that direct cell-to-cell contact by MACB resulted in the specialized metabolism of *S*. *lividans*. Supporting this, a mycolic acid-deficient mutant of *Corynebacterium glutamicum* (*pks13*::*Km* ^*r*^ *)* was found to have lost its inducing ability [3]. The results of mycolic acid-deficient mutant testing further suggested that the mycolic acid components, which exist in the outer membrane, may play important roles in the induction of pigments by *S*. *lividans*.

In this study, to test whether *Streptomyces* responds to the direct interaction of MACB cells, we focused on the mycolic acid component of MACB cell membranes. We prepared dead cells of MACB by formaldehyde-fixation and ^60^Co γ-irradiation. Three MACB were used for experiments: *Rhodococcus erythropolis* PR4, *Rhodococcus opacus* B4, and *T*. *pulmonis*. After conservation of cell shape and mycolic acid components was confirmed, the induction of pigments production by dead, intact cells were compared with those exerted by live cells. At the end of the study, the morphology of interacting bacteria in combined-culture was investigated by scanning electron microscopy (SEM).

## Materials and Methods

### General

*Streptomyces lividans* TK23 was used as an indicator strain of two pigments (RED and ACT) production. The three MACB; *Tsukamurella pulmonis* TP-B0596 [3], *Rhodococcus erythropolis* PR4 (NBRC 100887), and *Rhodococcus opacus* B4 [39], were used as inducer strains. TSB medium (Trypto-Soya Broth, Nissui) was used for seed culture of all strains. A3M or YGGS medium were used for production culture of pigments (RED and ACT) by *S*. *lividans*, and observation of cell-cell interaction by SEM. A3M medium contains; 0.5% glucose, 2% starch, 2% glycerol, 1.5% pharmamedia, 0.3% yeast extract, 1% HP-20, adjusted to pH 7.2. To prepare A3M agar medium, 1.8% agar was added and HP-20 was removed from A3M medium. YGGS medium contains; 0.5% glucose, 2% starch, 2% glycerol, 0.3% yeast extract, adjusted to pH 7.2. To prepare YGGS agar medium, 1.8% agar was added.

### Formaldehyde fixation of MACB

*T*. *pulmonis*, *R*. *erythropolis*, and *R*. *opacus* were cultured in K-1 flasks containing 100 ml of TSB (Trypto-Soya Broth, Nissui) medium for 3 days at 30°C, 200 rpm until fully grown. Cells were collected by centrifugation (3000 ×*g*, 10 min, 4°C), packed in an autoclaved dialysis membrane (20 ml cell suspension), and soaked in neutralized formaldehyde (1% final concentration, Wako) in TSB medium (1 L). After 3 hours of treatment, formaldehyde was removed by dialysis with TSB medium (1 L) 9 times. Death of fixed bacteria were confirmed by inoculating the fixed cells which were originally from 2 ml culture (>10^8^ cfu) on TSB agar plates.

### Sample preparation for SEM analyses

Sample preparation for SEM analyses followed the procedure as previously reported in all the experiments conducted [40]. For example, to observe the cell shape, the fixed cells and non-fixed live cells from the same culture batch were spotted on water agar, and further treated with 2% OsO_4_ (Wako) vapor overnight. The OsO_4_ treated cells were sputter-coated with Pt/Pd (E-1030, Hitachi, Japan), and observed by scanning electron microscopy (S-4000, Hitachi, Japan). Non-formaldehyde treated cells were used as control to compare the cell shape.

### ^60^Co γ-irradiation of MACB

*T*. *pulmonis*, *R*. *opacus*, and *R*. *erythropolis* were cultured in K-1 flasks containing 100 ml of YGGS medium for 3 days at 30°C at 200 rpm until fully grown. Cells were collected from 800 ml of culture by centrifugation (3000 ×*g*, 10 min, 4°C), and washed with fresh YGGS medium twice. Collected cells were suspended in 60 ml fresh YGGS medium, and divided into six 15-ml conical tubes. Cells were irradiated with ^60^Co γ rays at the Food Irradiation Facility, Japan Atomic Energy Agency (Gunma, Japan). Irradiation doses were 5, 10, 20, 30 or 40 kGy that were regulated by changing the distance of the samples from the γ ray source. Death of γ-irradiated bacteria were confirmed by inoculating the irradiated cells which were originally from 2 ml culture (>10^8^ cfu) on TSB agar plates.

To confirm there are no damage to the cells by γ-irradiation, γ-irradiated cells and control live cells from the same culture batch were spotted on water agar and treated with 2% OsO_4_ vapor overnight. The OsO_4_ treated cells were sputter-coated with Pt/Pd for SEM observation. Non-irradiated cells were used as control to compare the cell shape.

### Extraction and TLC analyses of mycolic acids from the dead and live cells

Total lipids from *T*. *pulmonis*, *R*. *opacus*, and *R*. *erythropolis* were hydrolyzed, esterified, and extracted from live cells, formaldehyde-fixed cells, and γ-irradiated cells which were originally from the same culture volumes (approximately 0.3 g in dry cell weight). Total lipids were prepared by a method described previously [41]. Briefly, 2 ml of 10% KOH in methanol were added to the lyophilized cells, and heated at 100°C for 2 h. A 0.6 ml volume of 6 N HCl were added to acidify the solvent and the hydrolyzed fatty acids were extracted by 2 ml of hexane, twice. The hexane layer was dried *in vacuo* and 2 ml of BMS (benzene: methanol: sulfuric acid = 10: 20: 1) were added to the fatty acid extracts, and heated at 100°C for 2 h. One milliliter of water was added to the solution and fatty acid methylesters were extracted by 2 ml of hexanes twice. Methylesterified fatty acids were dissolved in 300 μl of CM (chloroform: methanol = 2: 1), and 2 μl were spotted on TLC. TLC was developed by HE (hexane: ethyl acetate = 19: 1, twice) and visualized by CAM (cerium-ammonium-molybdate). Mycolic acid methylesters appeared as blue spots.

### MALDI spiral-TOF mass spectrometry of mycolic acids from the dead and live cells

Matrix-assisted laser desorption ionization time-of-flight (MALDI TOF) mass spectra were collected on a JMS-S3000 MALDI spiral-TOFMS (JEOL, Tokyo, Japan) with a flight length of ca. 17 m, and eight cycles of the spiral trajectory to achieve high mass-resolving power. Ions generated by irradiation with a 349-nm Nd:YLF laser were accelerated at 20 kV. The settings of delay time and grid voltage were optimized to maintain ΔM = 0.02–0.03 Da, at full width at half maximum over the range of *m/z* 400–1300. Mass calibration was made with a poly(methylmethacrylate) standard (peak-top MW, *M*p = 625 or *M*p = 1310) purchased from Polymer Laboratories (Church Stretton, UK). Three mass spectra for each sample were collected and the mass spectral data were processed with Polymerix software (Sierra Analytics, Modesto, CA, USA).

### Test for pigment induction by dead and live cells on solid medium

#### Formaldehyde-fixed cell assays

Approximately 10^8^ cfu spores of *S*. *lividans* were applied on A3M agar (about 3 × 0.3 cm). Fixed cells (originally from 10 ml culture) of MACB were spotted on agar medium and semi-dried by air. For the control, 2 μl of live cells of MACB which were from the same cultured batch for fixed cells preparation and stored at 4°C were applied. The agar plates were incubated at 30°C during the observation. The experiments were repeated three times, and the plates were checked for more than 10 days.

#### γ-irradiated cell assays

Approximately 10^8^ cfu spores of *S*. *lividans* in 2 μl were spotted on YGGS agar. The γ-irradiated cells of MACB, originally from 2 ml culture, were spotted on agar medium and semi-dried by air. For the control, 2 μl of live cells which were from same cultured batch for γ-irradiated cells preparation and stored at 4°C were applied. The agar plates were incubated at 30°C during the observation. The experiments were repeated twice, and the plates were checked for more than 10 days.

### Test for pigment induction by dead and live cells in liquid medium

#### Formaldehyde-fixed cell assays

*S*. *lividans* was cultured in TSB medium for 3 days, and 1 ml of seed culture was used to inoculate 20 mL of A3M medium in 100 mL flask. The formaldehyde-fixed cells of MACB, originally from 40 mL of TSB culture, were added to the production medium. For the control, 200 μl of live cells of MACB which was cultured with 10 ml TSB in test tube at 30°C, 180 rpm, for 2 days, were added to the A3M medium at the same time. The flasks were shaken at 30°C at 180 rpm for 7 days.

#### γ-irradiated cells assays

*S*. *lividans* was cultured in TSB medium for 3 days, and 1 ml of seed culture was used to inoculate 10 mL of YGGS medium in a test tube. The γ-irradiated cells of MACB, originally from 20 mL of YGGS culture, were added to the production medium. For the control, 100 μl of live cells of MACB which was cultured with 10 ml TSB in test tube at 30°C, 180 rpm, for 2 days, were added to the YGGS medium at the same time. The test tubes were shaken at 30°C, 180 rpm for 7 days.

### Quantification of RED and ACT produced by *S*. *lividans*

RED and ACT were quantified by methods described previously [42]. Briefly, to quantify actinorhodins (ACT), 0.4 ml of 2 N KOH were added to 0.4 ml of cultures. After centrifugation (3000 ×*g*, 5 min), A_640_ of supernatants was determined by spectrometer, and the amounts were calculated by molar absorbance coefficient (ε_640_ = 25320). To quantify undecylprodigiosins (RED), cells from 0.4 ml cultures were collected by centrifugation (10000 ×*g*, 5 min). After drying cells *in vacuo*, 0.4 ml of MeOH were added. After shaking for 1 h, 0.4 ml of 1 N HCl in MeOH were added and centrifuged (3000 ×*g*, 5 min). A_530_ of supernatants was determined, and the amounts were calculated by molar absorbance coefficient (ε_530_ = 100500).

### SEM analyses of mixed-cultures with live or dead MACB cells

Cultures with live cells, fixed cells, or γ-irradiated cells were observed by SEM (S-4000, Hitachi). A 100 μl volume of cultures wasspotted on water agar (2%) and treated with 2% OsO_4_ vapor overnight (14–16 h). The OsO_4_ fixed culture cells were sputter-coated by Pt/Pd (E-1030, Hitachi). SEM imaging was performed multi-times, on independent combined-culture.

## Results

### MACB retained intact cell shapes by formaldehyde fixation

**Fig 1:**
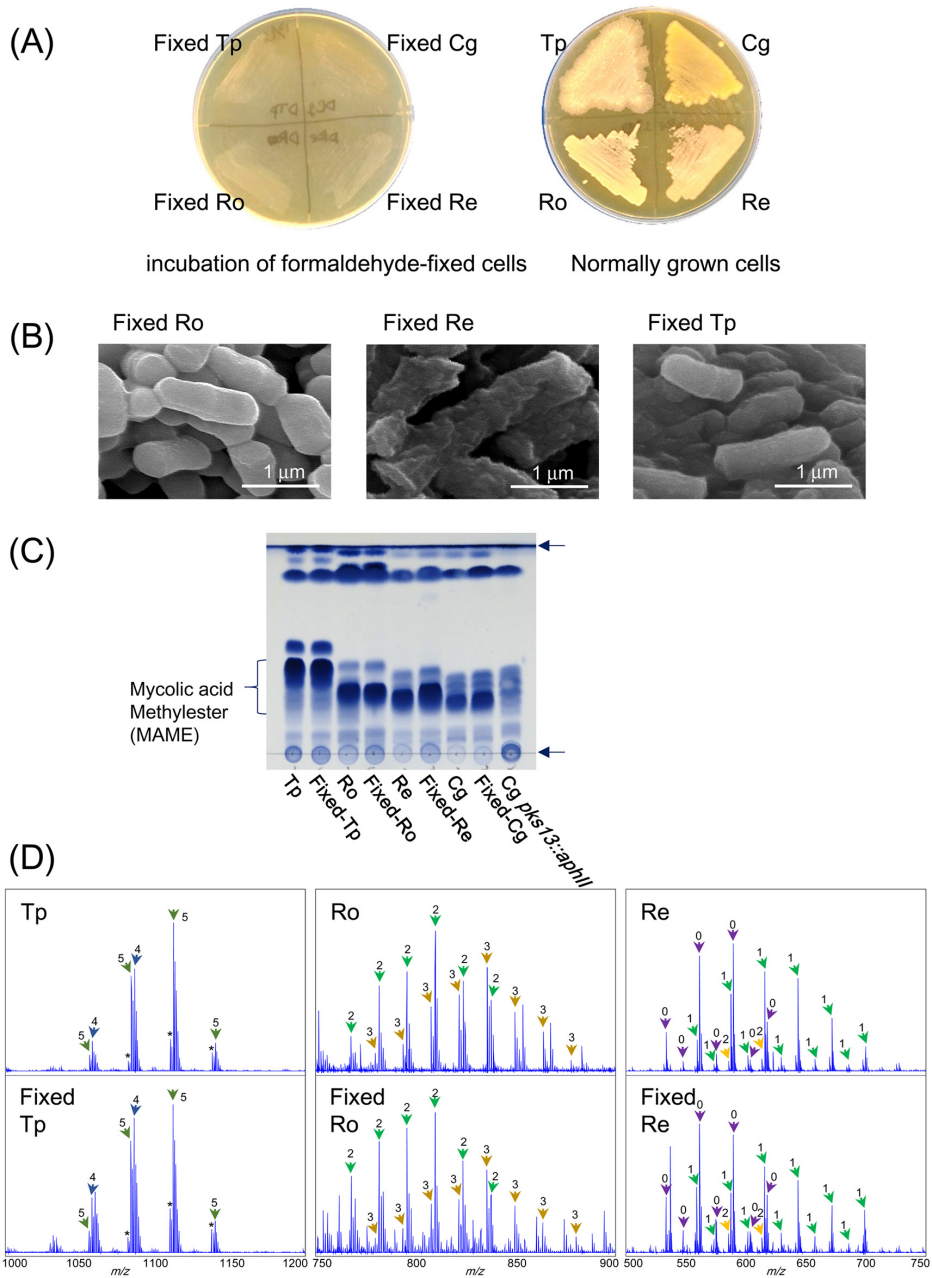
Conservation of cell shape and mycolic acids by formaldehyde-fixation. (A) Incubation of formaldehyde-fixed MACB on TSB medium. Formaldehyde-fixed MACB from 2 ml culture were inoculated on TSB agar medium and incubated at 30°C for 5 days. (B) SEM images of formaldehyde-fixed MACB. (C) TLC analysis of total fatty acid methylesters extracted from live or formaldehyde-fixed MACB. (D) MALDI mass spectra of mycolic acid methylesters from formaldehyde-fixed cells and non-fixed cells. Numbers on arrows represent (0) A-1 MA, (1) A-2 MA, (2) A-3 MA, (3) A-4 MA, (4) A-5 MA, and (5) A-6 MA (MA: mycolic acid). Definition of subtype (A-1 to A-6) are described in supporting information S1 Table. Abbreviations; Tp: *Tsukamurella pulmonis* TP-B0596, Ro: *Rhodococcus opacus* B4, Re: *Rhodococcus erythropolis* PR4, Cg: *Corynebacterium glutamicum* ATCC13869.

To produce the intact dead cells in the cell shapes and mycolic acid component, first we tried formaldehyde fixation. Formaldehyde fixation, which is a general cell fixation method for investigations in cell biology, enables the maintenance of cell shapes [43], by modification of protein/peptides by linking functional groups [44]. *R*. *erythropolis*, *R*. *opacus*, and *T*. *pulmonis* were treated with neutralized formaldehyde to kill the cells. Formaldehyde treated bacteria was not grown on agar medium which confirmed the death of cells (Fig 1A). Formaldehyde-fixed and non-fixed cells were observed by SEM, to compare their cell structure, and formaldehyde-fixed cells were shown to retain their cell shapes compared to the non-fixed cells of MACB (Fig 1B and S1 Fig).

### Mycolic acids were not damaged by formaldehyde-fixation

Total lipids from both fixed and non-fixed cells of *R*. *erythropolis*, *R*. *opacus*, and *T*. *pulmonis* were hydrolyzed, methylesterified, and extracted. Methylesterified total lipids were first analyzed by TLC (Fig 1C). Visualized spots of total lipids, including mycolic acids, showed almost identical patterns in TLC analysis, indicating the conservation of the lipid layer in the cell membrane. The extracted fatty acid methylesters were further analyzed by MALDI TOF mass spectrometry with a spiral ion trajectory (MALDI spiral TOF-MS) [45,46]. Mycolic acid fractions showed identical patterns between formaldehyde-fixed and non-fixed cells (Fig 1D).

### Properties of mycolic acids from three MACB strains

MACB biosynthesize diverse carbon chain length and different saturation types of mycolic acids. Generally known carbon chain lengths of mycolic acids are: *Corynebacterium*, C_20_ to C_38_; *Rhodococcus*, C_34_ to C_52_; *Tsukamurella*, C_64_ to C_78_; and *Mycobacterium*, C_60_ to C_90_ [47]. Using MALDI TOF-MS, we identified that *R*. *erythropolis* PR4 contains mycolic acids with carbon chain length of C_32_-C_44,_ consisting of subtype A-1 and A-2 (see supporting information S1 Table for subtype definition). *T*. *pulmonis* TP-B0596 contained mycolic acids with carbon chain length of C_72_-C_76,_ consisting of subtype A-5 and A-6. Interestingly, *R*. *opacus* B4 contained mycolic acids with carbon chain length of C_50_-C_56,_ consisting of subtype A-3 and A-4. Mycolic acids of *R*. *opacus* possessed relatively longer chain length than previously reported for *Rhodococcus* species [47]. To test the effect of mycolic acids, we obtained MACB strains which possessed different patterns of mycolic acids.

### Formaldehyed-fixed MACB did not induce pigments by *S*. *lividans*

**Fig 2:**
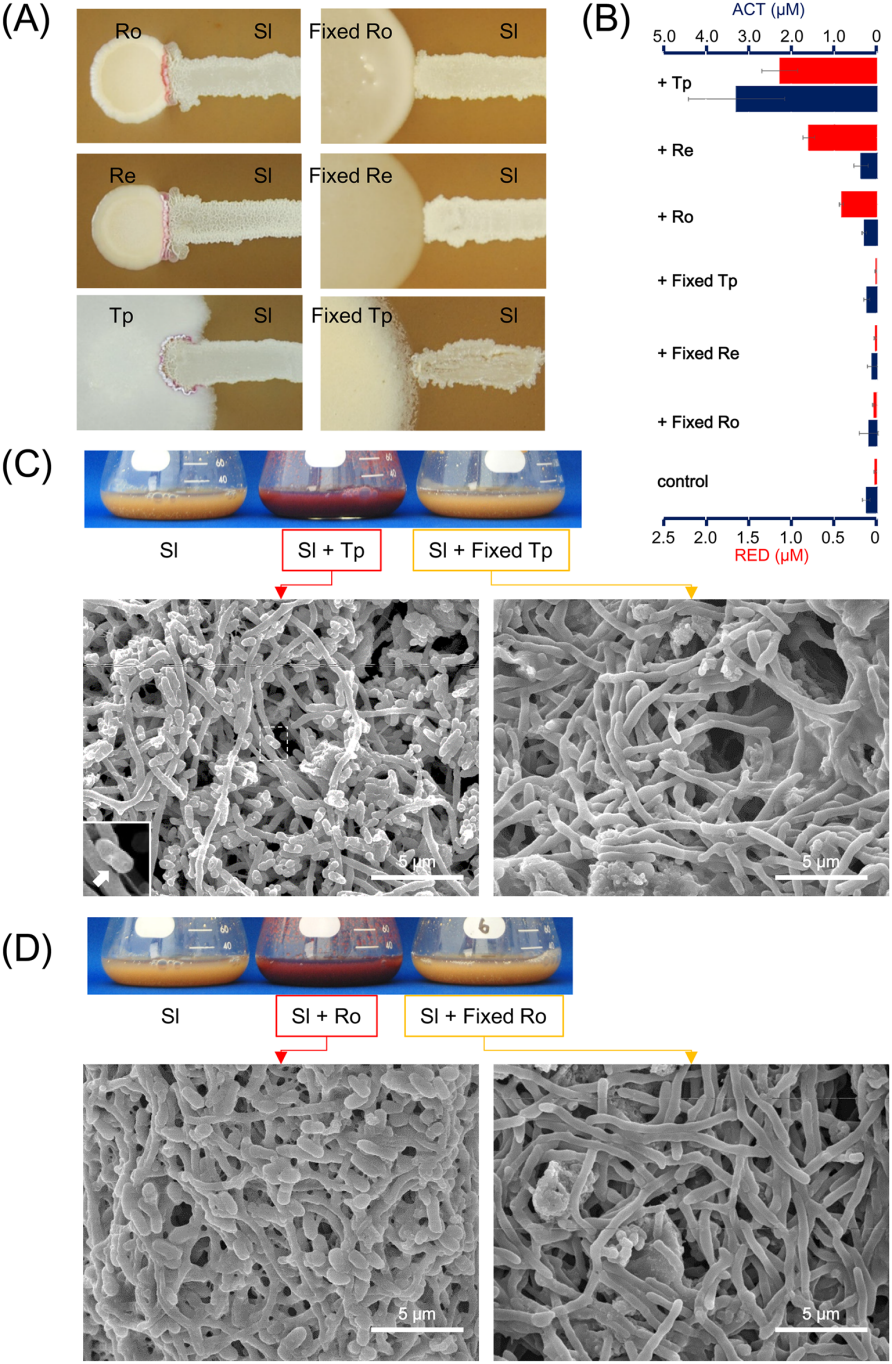
Pigments inducing effect by formaldehyde-fixed MACB. (A) Induction activity of formaldehyde-fixed MACB was tested on solid medium. Both bacteria were inoculated on A3M agar and incubated for 5 days at 30°C. (B-C) Induction activity of formaldehyde fixed MACB were tested in liquid medium culture. (B) Flask of mixed-culture and SEM images of mixed-culture of *S*. *lividans* and live *T*. *pulmonis* (left) or fixed *T*. *pulmonis* (right). The boxed image (left bottom) indicates the rod-shaped MACB. (C) Flask of mixed-culture and SEM images of mixed-culture of *S*. *lividans* and live *R*. *opacus* (left) or fixed *R*. *opacus* (right). Bacteria were cultured in A3M medium for 5 days at 160 rpm, 30°C. (D) Quantification of pigments (n = 3) in mixed-culture of formaldehyde fixed MACB or live MACB in A3M liquid medium. Red bars indicate the amount of RED (undecylprodigiosins), and blue bars indicate the amount of ACT (actinorhodins). Error bars indicate Mean ± S.D.

First, inducing effect were tested by agar medium. Fixed cells, from 2 ml of culture, were spotted on A3M agar medium, and *S*. *lividans* was grown on the side. For the control, live MACB culture was spotted. Live cells started to spread in 2–3 days, and the two bacterial genus started to interact directly. One day after the colonies contacted with each other, production of pigments by *S*. *lividans* were observed at the interface of the interacting colonies (Fig 2A). In contrast, *S*. *lividans* did not produced pigments at the interface of the fixed cells (Fig 2A).

Subsequently, inducing effect were tested by liquid medium. *S*. *lividans* and fixed cells from 20 ml culture were inoculated into the same A3M medium, and cultured. Enhanced production of RED and ACT was observed in combined-culture, by adding live MACB compared to the mono-culture of *S*. *lividans* (Fig 2B–2D). In contrast, no enhanced production of RED and ACT was observed by addition of fixed cells compared to the mono-culture of *S*. *lividans* (Fig 2B–2D). Both cultures were imaged by SEM for further analyses, as described in a later section (Fig 2B and 2C).

### MACB retained intact cell shapes by ^60^Co γ-irradiation

**Fig 3:**
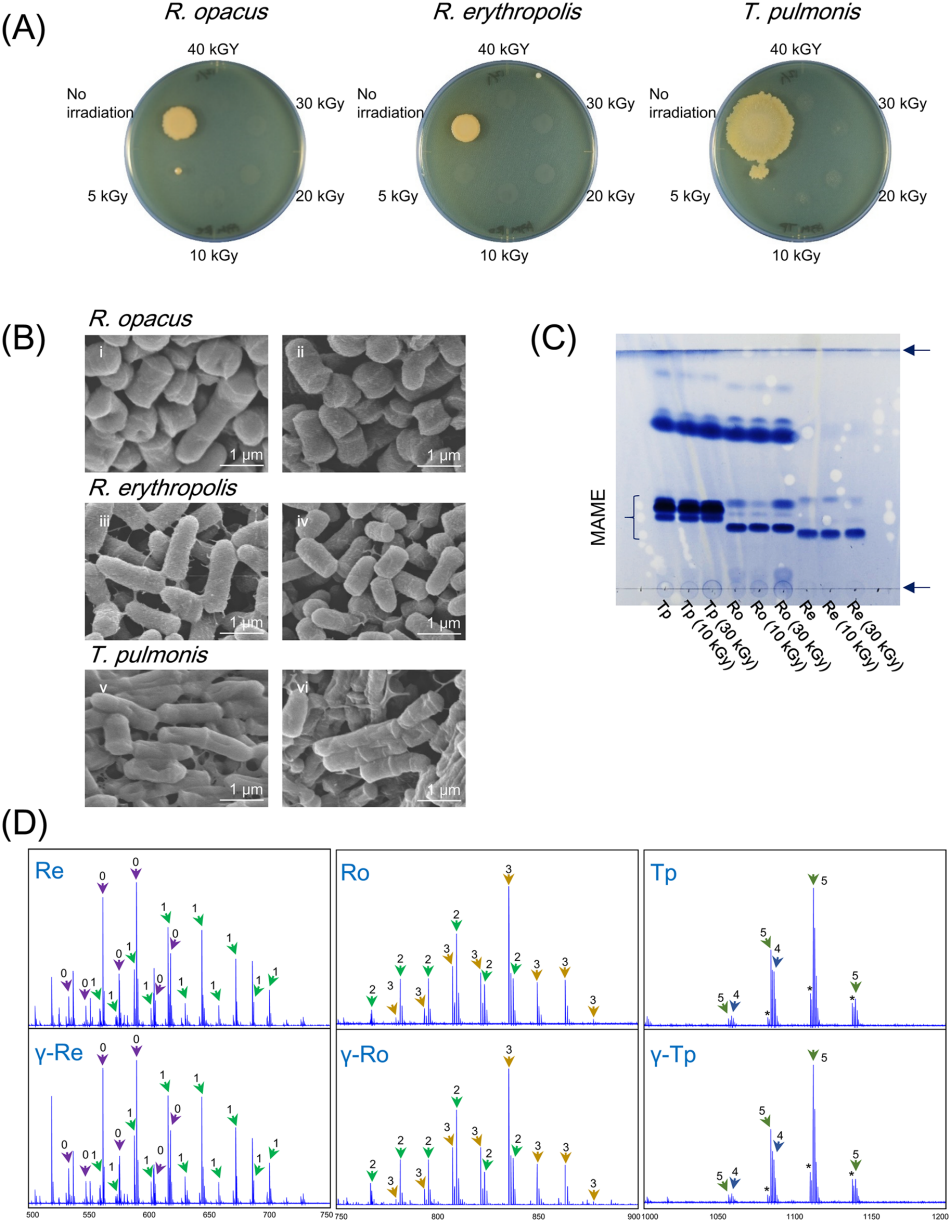
Conservation of cell shape and mycolic acids by γ-irradiation. (A) Incubation of γ-irradiated MACB on TSB medium. γ-irradiated MACB from 2 ml culture were inoculated on TSB agar medium, and incubated at 30°C for 5 days. (B) SEM images of γ-irradiated MACB. (i) non-irradiated cells of *R*. *opacus*. (ii) γ-irradiated (5 kGy) cells of *R*. *opacus*. (iii) non-irradiated cells of *R*. *erythropolis*. (iv) γ-irradiated (5 kGy) cells of *R*. *erythropolis*. (v) non-irradiated cells of *T*. *pulmonis*. (vi) γ-irradiated (5 kGy) cells of *T*. *pulmonis*. (C) TLC analysis of total fatty acid methylesters extracted from live or γ-irradiated MACB. (D) MALDI mass spectra of mycolic acid methylesters from γ-irradiated MACB and live cells. Numbers on arrows represent (0) A-1 MA, (1) A-2 MA, (2) A-3 MA, (3) A-4 MA, (4) A-5 MA, and (5) A-6 MA (MA: mycolic acid). Definition of subtype (A-1 to A-6) are described in supporting information S1 Table.

To confirm that indeed killing of MACB aborted inducing production of pigments by *S*. *lividans*, and mycolic acids on cell outer membrane have no effects, we tested an alternative method. ^60^Co γ-irradiation is a commonly used sterilization method by breaking genomic DNA and generateing reactive oxygen species (ROS) to modify biomolecules [48,49]. *R*. *erythropolis*, *R*. *opacus*, and *T*. *pulmonis* were cultured with YGGS medium for γ-irradiation. ^60^Co-irradiation was conducted at several different dose rates (1, 2, 4, 6 or 8 kGy/h) for 5 hours. Several growing cells were observed in the sample that received 5 kGy of irradiation (Fig 3A); however, we did not observe any growing cells in all the samples that received >10 kGy irradiation (Fig 3A). We chose the 10 kGy irradiated samples which were the minimum dose of irradiation to kill MACB completely in this experiment for further tests. The γ-irradiated (10 kGy) and non-irradiated cells were observed by SEM to show that cell shapes were not disrupted by the 10 kGy of irradiation (Fig 3B).

### Mycolic acids were not damaged by ^60^Co γ-irradiation

Total lipids from both γ-irradiated and non-irradiated cells of *R*. *erythropolis*, *R*. *opacus*, and *T*. *pulmonis* were analyzed by TLC. Visualized total lipids, including mycolic acids, showed almost identical patterns on TLC analysis, indicating the conservation of the lipid layer in the cell membranes (Fig 3C). The extracted fatty acid methylesters were further analyzed by MALDI spiral TOF-MS to show that mass spectra of the mycolic acids from γ-irradiated cells showed identical patterns, compared with non-irradiated cells (Fig 3D).

### γ-irradiated MACB did not induce pigments by *S*. *lividans*

**Fig 4:**
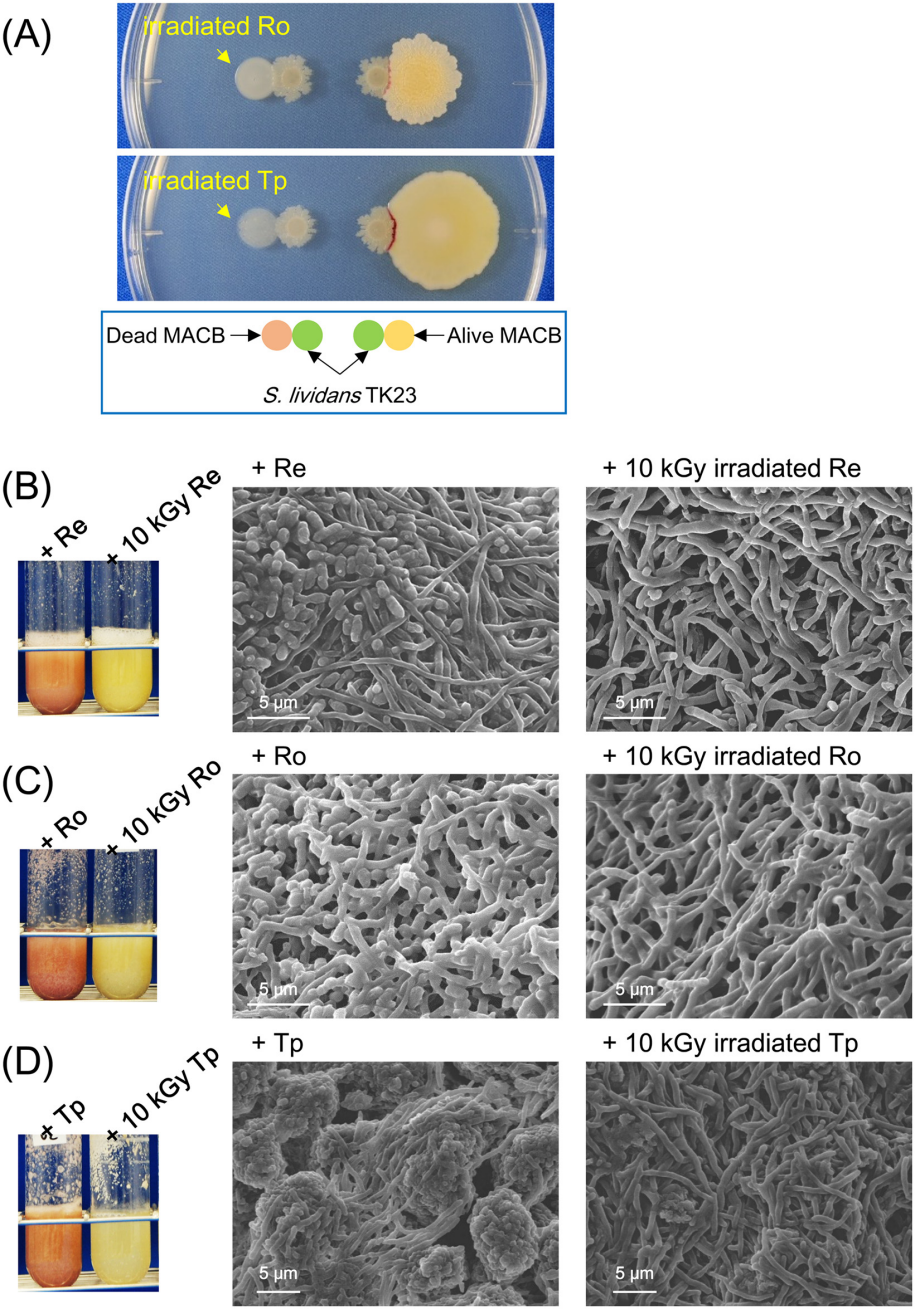
Pigments inducing effect by γ-irradiated MACB. (A) Induction activity of γ-irradiated MACB was tested on solid medium culture. Both bacteria were inoculated on YGGS agar and incubated for 5 days at 30°C. (B-D) Induction activity of γ-irradiated MACB were tested in liquid medium. SEM images were obtained from mixed-cultures in YGGS medium (5 days at 180 rpm, 30°C): (B) *S*. *lividans* with live *R*. *erythropolis* (left) or γ-irradiated *R*. *erythropolis* (right). (C) *S*. *lividans* with live *R*. *opacus* (left) or γ-irradiated *R*. *opacus* (right). (D) *S*. *lividans* with live *T*. *pulmonis* (left) or γ-irradiated *T*. *pulmonis* (right).

The γ-irradiated cells from 2 ml culture were spotted on YGGS agar medium, and *S*. *lividans* was grown on the side. For the control, live MACB cells were spotted. Spotted live cells started to spread, and the colonies of two bacterial genus started to interact directly in 2–3 days. A day after direct contact of colonies by the two genus, pigments production by *S*. *lividans* was observed in the area where both colonies contact (Fig 4A). In contrast, no pigment production was observed at the area where colonies of γ-irradiated cells and *S*. *lividans* were attached.

*S*. *lividans* and γ-irradiated cells from 20 mL culture were inoculated into YGGS medium and cultured. Enhanced production of the red pigment was observed by combined-culture adding live MACB (Fig 4B–4D). In contrast, no enhanced production of red pigments was observed by addition of γ-irradiated cells (Fig 4B–4D).

### Co-aggregation was observed by mixing live MACB

**Fig 5:**
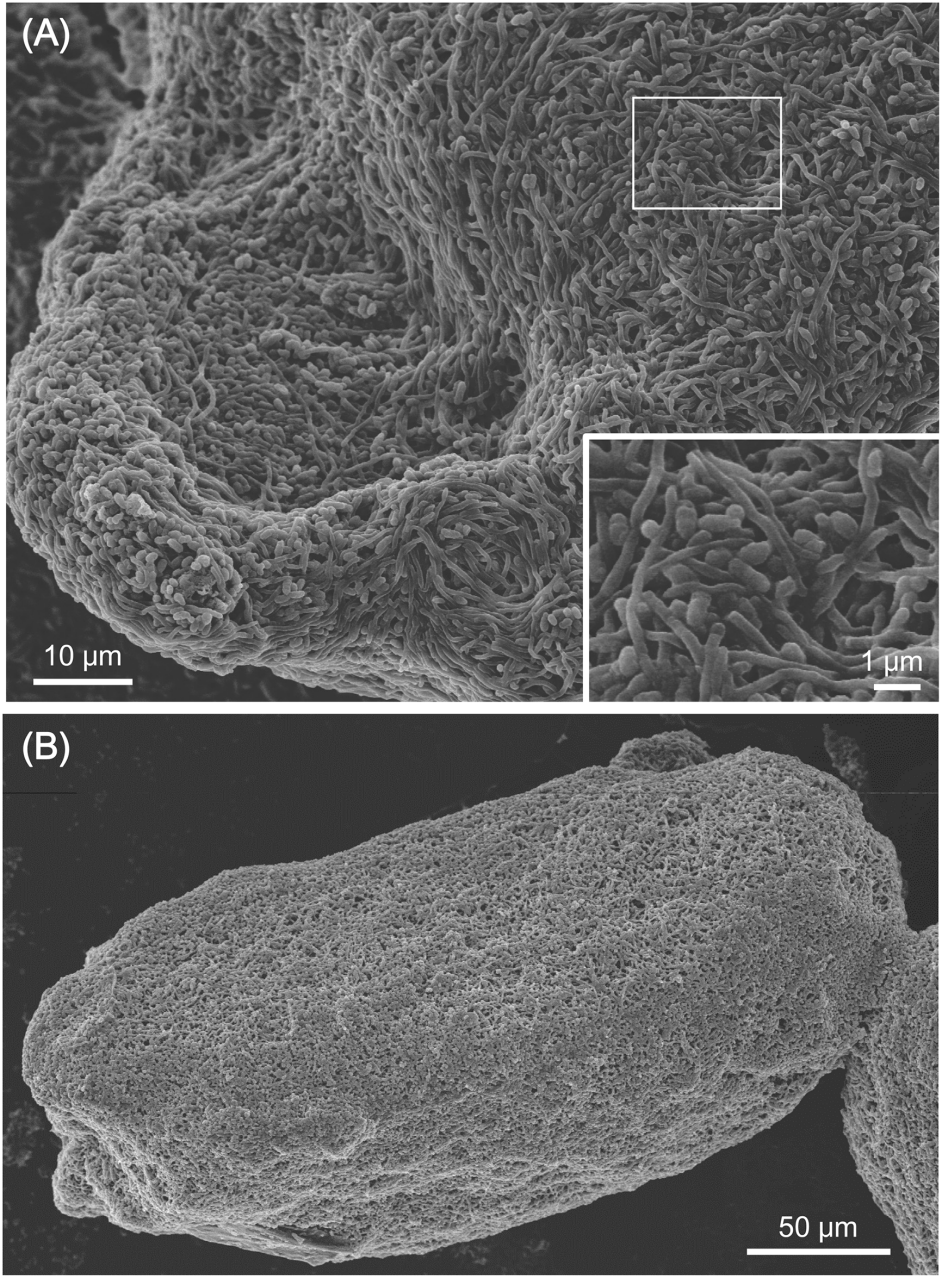
Co-aggregation of *S*. *lividans* and MACB observed using SEM. (A) Co-aggregation of *S*. *lividans* and live *R*. *erythropolis*. Adhesion of rod-shaped cells of *R*. *erythropolis* was observed entirely on pellets of *S*. *lividans* formed in liquid culture. (B) Co-aggregaton of *S*. *lividans* and live *R*. *opacus*. Adhesion of rod-shaped cells of *R*. *opacus* were observed entirely on pellets of *S*. *lividans* formed in liquid culture. Bacteria were cultured in YGGS medium for 5 days at 180 rpm, 30°C.

Cell-cell interactions with formaldehyde fixed cells and live cells were compared by SEM imaging. Combined-culture of *S*. *lividans* with *T*. *pulmonis* or *R*. *opacus*, mixed-culture of *S*. *lividans* with fixed *T*. *pulmonis* or fixed *R*. *opacus*, which were cultured in A3M medium, were observed by SEM. *Streptomyces* appeared as filamentous cells, and MACB appeared as rod-shaped cells. In the mixed-culture of *S*. *lividans* and live *T*. *pulmonis* or *R*. *opacus*, the MACB showed significant adhesion to the mycelia of *S*. *lividans* (Fig 2B and 2C). In contrast, in the mixed-culture with formaldehyde-fixed dead cells, the cell adhesions which were observed in mixed-culture with live MACB cells were not observed (Fig 2B and 2C). Subsequently, to investigate the cell attachment interactions by live MACB, mixed-culture of *Streptomyces* with γ-irradiated dead cells and live cells were compared. Consistently, in the mixed-culture of *S*. *lividans* with live MACB, the MACB showed significant adhesion to the mycelia of *S*. *lividans* (Fig 4B–4D) forming co-aggregation (Fig 5A and 5B). In contrast, when mixed with γ-irradiated dead cells, the co-aggregation that had been observed in combined-culture with live MACB cells was not observed (Fig 4B–4D). To observe the distribution of the attachments of MACB to the mycelial pellets / *clumps formed by S*. *lividans*, SEM images were taken at low magnification. *The MACB appeared to attach entirely on the* pellets / *clumps formed by S*. *lividans*, *to build co-aggregation forms* (Fig 5A and 5B).

## Discussion

In order to elucidate that cell structure with intact mycolic acids on cell membranes of MACB is required for the induction of cryptic pigments (ACT and RED) by *S*. *lividans*, dead cells of MACB were prepared by formaldehyde fixation and ^60^Co γ-irradiation. First, conservation of cell shape of dead MACB was verified by SEM. The SEM observation showed that observable damage was not found in both cells prepared by formaldehyde fixation or ^60^Co γ-irradiation. Then, mycolic acids in the cell membrane of the dead cells were analyzed by TLC and MALDI TOF-MS. Extracted total lipids from same amount of dead cells and live cells were compared using TLC. TLC analyses showed no significant change of fatty acids components in samples from both live or dead cells. Further, the MALDI mass spectra of mycolic acids were compared between the dead and live cells, which showed identical patterns of mycolic acid components in the dead cells produced by the two methods described, as compared with those of live cells. The comparison of MALDI mass spectra and TLC analyses clearly indicated that qualitatively mycolic acids were intact, and no significant quantitative change of mycolic acids components were occurred by treatment of formaldehyde fixation and ^60^Co γ-irradiation, which can be judged from the size of the visualized spots on TLC.

By using these dead intact cells, we tested induction of RED and ACT by *S*. *lividans*. As a result, dead cells produced by both formaldehyde fixation or ^60^Co γ-irradiation did not induce any pigment production by *S*. *lividans*. The results of activity assays, in both solid and liquid media, showed that the cell structure with intact mycolic acids is not sufficient for the induction of pigments in *S*. *lividans*, which indicate that additional or alternative factor(s) are necessary.

To further investigate the interactions of *S*. *lividans* and MACB, since dead cells were unable to induce pigments production by *S*. *lividans*, the interactions between *S*. *lividans* and live or dead MACB cells were investigated using SEM. Interestingly, we found significant adhesion of live MACB to *Streptomyces* mycelium in combined-culture. The two different bacteria were forming co-aggregation through the cell pellets/clumps of *S*. *lividans*. *S*. *lividans* normally grow to form mono-strain pellets/clumps in liquid culture, therefore MACB was likely to adhere on the surface of *S*. *lividans* mycelium during the culture. These adhesive interactions between *Streptomyces* species and other bacteria have been shown for *Streptomyces griseus* and *Bacillus amyloliquefaciens* with extensive network of fimbriae-like structures when forming a mixed-biofilm structure, compared with the single-species biofilm [50].

When we observed the mixed-culture with dead MACB cells, in contrast to the adhesive interaction in the mixed-culture with live cells, co-adhesion of dead MACB to the mycelium of *S*. *lividans* was not observed, although we added the amount of dead cells that is estimated to be more than equivalent of the full growth of MACB. This indicates that adhesion properties in MACB are disrupted by the killing processes.

Multispecies biofilms can be organized in three different ways: separated, layered, or co-aggregated states [51]. In combined-culture, SEM observation indicated that the two genetically distinct bacteria formed co-aggregations. Such co-aggregations of intergeneric bacteria are commonly observed in oral biofilms, and within such multispecies biofilms, intergeneric co-aggregation partnerships show specific relationships with the membrane protein involved [52]. As both *Streptomyces* and MACB are *non-flagellated* bacteria, some factors, such as chemical charges [53], and pili or specific membrane proteins [54], can be predicted to mediate the cell attachment between *S*. *lividans* and MACB. One suggested factor was the hydrophobicity of the mycolic acid layer, which is proposed to be responsible for adhesion of MACB to a material surface [53,55]; however the dead cells conserved with the lipid rich outer membrane were not observed to adhere on the mycelium of *S*. *lividans* in mixed-culture, indicating low adhesion possiblily. Alternatively, bacterial cell membrane proteins, known as adhesins, are the most commonly known cell component to promote co-adhesion in inter-generic bacteria [52], which could possibly be involved in the co-adhesion of *Streptomyces* and MACB.

In conclusion, we successfully produced dead cells of conserved cell shape with intact mycolic acids of MACB by formaldehyde fixation and γ-irradiation. Killing of MACB was found to eliminate induction of specialized metabolites by *S*. *lividans*. In addition to the results indicating that the attachment of mycolic acid membranes was not the sole inducing factor for pigments production by *S*. *lividans*, we showed that *S*. *lividans* and live MACB co-aggregate when pigments were induced, whereas no such co-aggregation formed with dead cells which did not induced production of pigments. Although many possibilities in activation of pigments production caused by MACB can be considered, the continuous attachment of MACB on *S*. *lividans* mycelium was proposed as a trigger that alters the specialized metabolism in *S*. *lividans*.

## Supporting Information

S1 Fig Comparison of cell shape of formaldehyde-treated MACB and non-treated MACB. The white bars in the SEM images indicate the scales of 3 μm.
(TIF)

S1 Table Structures of mycolic acid (MA). (PDF)
