# Supplementary figures and images for: Killing of Mycolic Acid-Containing Bacteria Aborted Induction of Antibiotic Production by Streptomyces in Combined-Culture
Source: PLoS One. 2015 Nov 6;10(11):e0142372. doi: 10.1371/journal.pone.0142372 (PMC4636228; doi:10.1371/journal.pone.0142372)

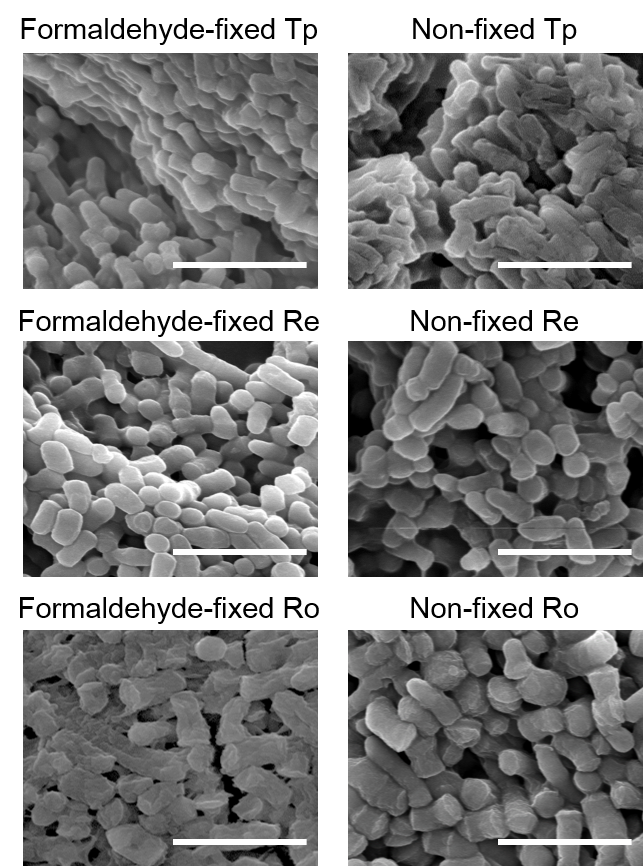

Supplement: S1 Fig — The white bars in the SEM images indicate the scales of 3 μm. (TIF) [file pone.0142372.s001.tif]
