# Supplementary material for: Killing of Mycolic Acid-Containing Bacteria Aborted Induction of Antibiotic Production by Streptomyces in Combined-Culture
Source: PLoS One. 2015 Nov 6;10(11):e0142372. doi: 10.1371/journal.pone.0142372 (PMC4636228; doi:10.1371/journal.pone.0142372)
Supplement: S1 Table — (PDF) [file pone.0142372.s002.pdf]

S1 Table. Structures of mycolic acid (MA).

subtype		structure		
Saturated MA	A-1	$C_3H_6O_3$	+	$(CH_2)_n$
Monoenoic MA	A-2	$C_3H_4O_3$	+	$(CH_2)_n$
Dienoic MA	A-3	$C_3H_2O_3$	+	$(CH_2)_n$
Trienoic MA	A-4	C_3O_3	+	$(CH_2)_n$
Tetraenoic MA	A-5	C_4O_3	+	$(CH_2)_n$
Pentaenoic MA	A-6	C_5O_3	+	$(CH_2)_n$
